# Evaluation of Ultrasonic Vibration-Assisted Grinding in Multi-Process Profile Grinding of K4002 Nickel-Based Superalloy Blade Tenons

**DOI:** 10.3390/ma18071437

**Published:** 2025-03-24

**Authors:** Yang Cao, Yun He, Fei Liu, Benkai Li, Zheng Li, Xiaobo Guo, Zhangquan Lv

**Affiliations:** 1 School of Mechanical and Aviation Manufacturing Engineering, Anyang Institute of Technology, Anyang 455000, China; caoy2025@foxmail.com (Y.C.); heyun296415@foxmail.com (Y.H.); liufei07104517@163.com (F.L.); lvzq2016@163.com (Z.L.); 2 School of Mechanical and Automotive Engineering, Qingdao University of Technology, Qingdao 266520, China; 3 School of Mechanical Engineering, Zhengzhou University of Aeronautics, Zhengzhou 450046, China; lz1982131@163.com

**Keywords:** blade tenon grinding, intermittent cutting behavior, grinding force, grinding temperature, surface integrity

## Abstract

The fir-tree blade tenon is an important connection part of the turbine blade; its machining quality directly affects the life and power of the aeroengine. At present, the machining of the blade tenon requires multiple profile grinding processes. This study highlights the whole profile of the grinding processes of K4002 nickel-based superalloy blade tenons in ultrasonic vibration-assisted grinding (UVG). A probability superposition method was utilized to calculate the undeformed chip thickness and contact rate considering the random distribution of the abrasive grains and the overlap of the grinding trajectories. Subsequently, the grinding force, grinding temperature and surface integrity of the blade tenons in conventional grinding (CG) and UVG were investigated. The results indicate that the ultrasonic vibration causes intermittent cutting behavior which can reduce the contact rate to 0.6 at most. The grinding force, grinding temperature and surface integrity are deeply affected by the fir-tree shape of the blade tenon. The maximum grinding force occurs at the start of the full contact stage; surface burnout easily occurs in the middle top area of the blade tenon. Compared to CG, the use of UVG leads to an average reduction in the grinding force and temperature by 20% and 23%, respectively, improving the surface burnout of the K4002 superalloy.

## 1. Introduction

The turbine blade is a key part of the aeroengine and plays an important role in generating power for the aircraft [1]. The blade tenon, located at the root of the turbine blade, has a specifical fir-tree shape which connects the turbine blade to the turbine disc, tightly and precisely. Behind this, the blade tenon usually needs high-quality machining. At present, profile grinding is the main method for the machining of the blade tenon [2,3,4]. However, profile grinding is now faced with the problems of large force, high temperature and easy surface burnout due to the use of difficult-to-cut nickel-based superalloy materials [5,6,7]. Even though a lot of advanced surface grinding technologies have been reported, the investigation on profile grinding of the blade tenon is still rare.

Profile grinding is more complicated than surface grinding as the grinding width, contact area shape and abrasive wheel wear are changing during the machining process [8,9,10]. Therefore, the grinding temperature is differently distributed in the different positions of the blade tenon. Li et al. [11] divided the fir-tree shape of the turbine disc slot into 18 areas in profile grinding. The grinding temperature at the slot peak was 30% higher than that at the slot bottom due to the uneven heat flow density distribution. Similarly, Yin et al. [12] found that the thermal damage likely occurs at the convex teeth of the blade tenon. The use of a CBN abrasive wheel can effectively reduce the grinding temperature, improving the thermal damage. It can be found that more and more attention is paid to profile grinding technology. However, these studies mainly focus on the performance of one specific grinding process under different parameters. The whole machining of the blade tenon involves many grinding processes. All the grinding processes are interactional in the case of grinding force, temperature and surface quality, which are rarely studied.

Ultrasonic vibration-assisted grinding (UVG) is a promising composite machining method for the difficult-to-cut alloys [13,14]. Zhao et al. [15] found that the temperature was reduced by 39.1% when the ultrasonic vibration was active in the profile grinding of a γ-TiAl blade tenon. Miao et al. [16] noticed that the use of ultrasonic vibration assistance in the high-speed grinding of nickel alloy led to a superiority of the material structure gradient, resulting from the coupled effect of the high grinding speed and severe ultrasonic impact. Zhou et al. [17] pointed out that ultrasonic vibration could significantly reduce the friction force between the abrasive grain and workpiece; because the ultrasonic vibration caused the improvement of the adhesive effect in the material removal process. Moreover, the effect of UVG was more significant under the larger undeformed chip thickness or lower grinding speed. Naskar [18] found that a 20–100% improvement in compressive residual stress was obtained when ultrasonic vibration was used in the high-speed grinding of Ti-6Al-4V. These investigations indicate that UVG has the advantages of low grinding force, low temperature and good surface integrity under the ideal specific processing parameters and conditions. But the effect of UVG in the actual multi grinding process is insufficiently investigated.

The undeformed chip thickness and contact rate are important indexes in UVG; they determine the grinding force, grinding temperature and surface integrity [19,20,21]. Today, a lot of theoretical models have been established between the undeformed chip thickness and the grinding force and temperature. But, the calculation of the undeformed chip thickness is difficult in UVG, especially when the random distribution of abrasive grains and the overlap of grinding trajectories are considered. Pass-by-pass simulation is a method to solve the problem [22,23]. But the simulation is computationally intensive due to the large number of abrasive grains on the wheel surface. Liu et al. [24] established a dynamic grinding force model considering the randomized grain geometric characteristics. The amplitude of the force fluctuation and the average force value were obtained with a maximum error of 5.1%. In some cases, the equivalent undeformed chip thickness is utilized in the force model for simplifying [25,26]. Zhang et al. [27] established a grinding force model for UVG using the maximum undeformed chip thickness. The study indicated that the ultrasonic amplitude and grinding depth were independent from the maximum undeformed chip thickness. Li et al. [19] found that the maximum undeformed chip thickness related to the elasticity modulus of the abrasive grain and workpiece material. The material of the high elastic modulus could lead to a reduction in undeformed chip thickness. However, the errors cannot be ignored due to the lack of consideration of nonuniform grain size and grain distribution. A convenient and accurate method for calculating the undeformed chip thickness is needed.

This study highlights the entire grinding processes of blade tenons using UVG. First, the experimental details are introduced, involving two kinds of processing parameters and two kinds of grinding methods (conventional grinding (CG) and UVG). Then, the contact rate in UVG is calculated on the basis of a novel probability superposition method. At last, the grinding force, grinding temperature and surface integrity in UVG and CG are discussed. The conclusions are then summarized.

## 2. Experimental Details

### 2.1. Experimental Device

**Figure 1:**
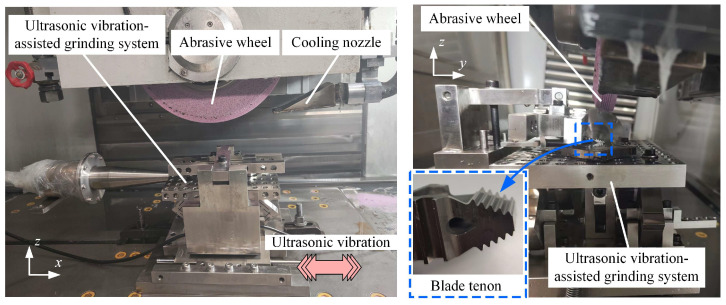
Ultrasonic vibration-assisted grinding experimental device.

The experiments were conducted on a worktable of a 3-axis profile grinder (Smart BD10, ELB, Viernheim, Germany) (Figure 1), with the experimental conditions provided in Table 1. A self-developed ultrasonic vibration system [28] for the blade tenon was positioned at the center of the grinder’s worktable. The ultrasonic vibration system was connected to a dedicated ultrasonic generator which output high-frequency electrical signals. The signals were converted into mechanical vibrations by the ultrasonic transducer. The ultrasonic vibration was mainly along the worktable infeed direction (*x*-axis in Figure 1).

The K4002 nickel-based superalloy turbine blade was clamped in the center of the ultrasonic vibration system; the blade tenon was exposed for machining. Abrasive wheels (WA/PA80/100F8V35m/sCF3) were utilized. The abrasive wheels had an original outer diameter of 400 mm and thickness of 30 mm. During the experiment, a diamond roller was employed to dress the grinding wheel. The dressing speed and infeed depth were set to 20 m/s and 0.3 µm/r, respectively, with a total dressing allowance of 0.2 mm. The grinding fluid consisted of 5% water-based emulsion (Syntilo 9954, Castrol, Wiltshire, UK), with maximum cooling pressure of 0.5 MPa. Prior to the experiment, the height of the grinding fluid nozzle was adjusted to ensure effective delivery of the fluid into the grinding area. Furthermore, the position of the coolant nozzle was linked to the radius of the grinding wheel, allowing automatic adjustment when the wheel was dressed, ensuring efficient coolant delivery to the grinding area for heat exchange.

### 2.2. Experimental Process

In the experiments, the amplitude of ultrasonic vibration at the workpiece was 5 μm. The workpiece of the blank blade tenon had a trapezoidal shape with total grinding allowance of 1.56 mm. Down grinding mode was utilized in the experiments for short grinding arc and low grinding temperature.

The experiments were divided into four groups based on parameter configurations, as listed in Table 2 and Table 3. The parameters of Group I and Group II had conservative material removal rate. The blade tenon was completed in six grinding processes. The grinding depth of these processes was 0.6 mm, 0.36 mm, 0.3 mm, 0.2 mm, 0.09 mm and 0.01 mm in turn. During the six grinding processes, the abrasive wheel was dressed twice using the dressing parameters in Table 1; one was before the third grinding process and the other was before the last grinding process. For comparison, CG was adopted in Group I, while UVG was adopted in Group II.

The parameters of Group III and Group IV were large material removal rates. The quantity of grinding processes was reduced to five, as listed in Table 3. The grinding depth was 0.75 mm, 0.45 mm, 0.24 mm, 0.11 mm and 0.01 mm in turn. Conversely, the abrasive wheel was dressed only once before the last grinding process. Group III used the CG method and Group IV used the UVG method.

For each experiment group, the abrasive wheel was firstly dressed by the roller dresser before the grinding process 1 for the same sharpness of abrasive grains at the experimental start. After the grinding process, the blade tenons were cleaned by alcohol solution using the ultrasonic cleaning method for 5 min. Then, the fine blade tenons were stored in dry containers. For every experiment group, the whole grinding was repeated three times. A total of twelve experimental results were processed and discussed.

**Figure 2:**
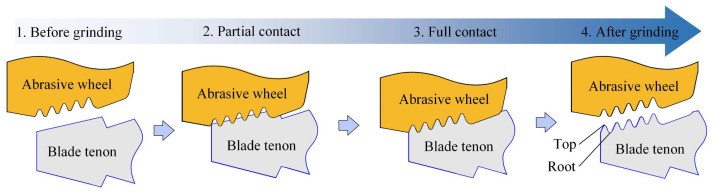
Partial contact and full contact stages in blade tenon profile grinding.

The blank blade tenon was casted in a trapezoidal shape, as shown in Figure 2. The grinding process can be categorized into partial contact and full contact stages with the decrease in machining allowance. When the abrasive wheel firstly contacts the blank blade tenon, the material removal occurs only in the root area of the tenon tooth, entering the partial contact stage. At this stage, the actual removed material is minimal due to the narrow grinding width, but the material removal rate is high due to the large grinding depth in process 1. When the cumulative grinding depth increases, the top of the tenon teeth contacts the abrasive wheel, causing the entire grinding wheel profile to engage with the workpiece surface, entering the full contact stage, and the grinding width reaches the maximum value.

### 2.3. Test Method

During the grinding, the force was tested using the dynamometer (9253B, Kistler, Winterthur, Switzerland). The force signals were subsequently processed by a signal amplifier (5080A, Kistler, Winterthur, Switzerland) and DynoWare software (Version 3.2.0.0, Kistler, Winterthur, Switzerland). The force signals at the stable grinding stage were chosen and were low pass filtered at a cutoff frequency of 5 Hz, yielding a stable average grinding force value. The test was repeated three times, and the final grinding force was the average value.

The grinding temperature was measured using semi-artificial thermocouples. The data acquisition card (USB-6211, NI, Austin, TX, USA) was utilized, with a sampling frequency of 2 kHz. Afterwards, the data were processed using LabVIEW software (Version 20.0, NI, Austin, TX, USA), and a low-pass filter with a cutoff frequency of 50 Hz was applied [29]. The relationship between grinding temperature *T* and voltage *U* was calibrated. The relationship can be described as follows:

(1) $$T=0.596U^{2}+8.766U+20.582.$$

The final grinding temperature was obtained by averaging the test results.

The ground surface roughness and surface topography were measured using confocal microscopy (S Neox, Sensofar, Gavà, Spain) with a sampling length of 0.8 mm. The surface topography was observed using an optical microscope (HK-7700, Rirox, Shanghai, China). For one blade, there are five tenon teeth on one side, as shown in Figure 1. For one blade tenon tooth, three random positions at the top and three random positions at the root were measured. Therefore, a total of 360 areas on the tenon surface were observed and measured in the whole experiment.

## 3. Results and Discussion

### 3.1. Intermittent Cutting Behavior in UVG

#### 3.1.1. Theoretical Modeling of Intermittent Cutting

Due to the use of ultrasonic vibration, the abrasive grains were periodically in contact with and separate from the workpiece surface, resulting in intermittent cutting behavior [30]. The parameters of contact rate *r*_c_ and contact length in UVG  *l*_u_ are usually used to evaluate the degree of the intermittent cutting behavior. They can be described as follows [31]:

(2) $$r_{c}=\frac{l_{u}}{l_{\mathrm{arc}}},$$

(3) $$l_{u}=\frac{v_{s}}{\pi f}(\frac{\pi }{2}-arcsin(1-\frac{a_{u}}{A})),$$

where *l*_arc_ denotes the length of the grinding arc, $l_{\mathrm{arc}}=\sqrt{2a_{p}R}$ [32], *a*_p_ denotes the grinding depth, *R* denotes the radius of the abrasive wheel and *a*_u_ denotes the undeformed chip thickness in UVG. According to Equation (2), the value of *r*_c_ is related to *a*_u_. In this study, the undeformed chip thickness in CG is described, at first, on the basis of an equivalent grinding zone model. Then, the value of *a*_u_ is obtained by considering the influence of the ultrasonic vibration based on the probability superposition method.

In UVG, the blade tenon vibrates along the *x*-axis at the ultrasonic frequency, while the abrasive grains rotate and the grinding machine worktable infeeds along the *x*-axis. Therefore, the motion of the abrasive grain is composed of three parts, namely, the grinding speed *v*_s_, worktable infeed speed *v*_w_ and the ultrasonic vibration, which could be expressed as follows:

(4) $$x=R\sin(\omega t+\alpha )+v_{w}t+A\sin(\frac{2\pi }{f}t),$$

(5) z=Rcos(ωt+α),

where ω denotes the angular speed of the abrasive wheel, $\omega =v_{s}/R$; $v_{s}$ and $v_{w}$ are the grinding speed and workpiece infeed speed, respectively; and α, t, A and f denote the initial angle of the abrasive grain, time, amplitude of ultrasonic vibration and ultrasonic frequency, respectively.

**Figure 3:**
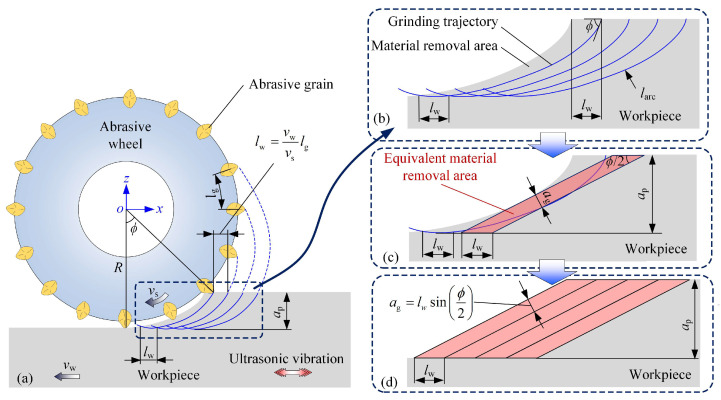
Illustration of material removal process in CG: (**a**) grinding trajectories; (**b**) actual material removal area; (**c**) equivalent material removal area of single abrasive grain; and (**d**) equivalent material removal areas of several abrasive grains.

According to Equations (4) and (5), the motion curve of the abrasive grain in CG can be obtained when the amplitude of the ultrasonic vibration equals 0, as illustrated in Figure 3a. The abrasive grains pass the grinding zone one by one to remove the material. The undeformed material removal area of a single abrasive grain is determined by the adjacent two grinding trajectories, as displayed in Figure 3b. To obtain the expression of the undeformed chip thickness in CG, the material removal area is simplified as a parallelogram. The parallelogram’s base length equals to the workpiece infeed distance $l_{w}$ between the interval of the abrasive grains. The parallelogram height equals to $a_{p}$, as illustrated in Figure 3c. As the contact angle of the abrasive grain is uniformly varied from ϕ to 0 during the material removal process, the average value of ϕ/2 is adopted as the tip angle of the parallelogram. Accordingly, the undeformed chip thickness in CG can be expressed as follows [25]:

(6) $$a_{g}=l_{w}\sin\left(\frac{\phi }{2}\right),$$

(7) $$l_{w}=\frac{v_{w}}{v_{s}}l_{g},$$

(8) $$\phi =\sqrt{\frac{2a_{p}}{R}},$$

(9) $$l_{g}=KM^{-1.4}\sqrt[{3}]{\frac{\pi }{32-S}},$$

where $a_{g}$ is the undeformed chip thickness in CG, $l_{g}$ is the distance between active abrasive grains and K, M and S are the constant, abrasive wheel size number and structure number, respectively. As a result, the undeformed chip thickness can be obtained as follows:

(10) $$a_{g}=KM^{-1.4}\frac{v_{w}}{v_{s}}\sqrt[{3}]{\frac{\pi }{32-S}}\sin\left(\sqrt{\frac{a_{p}}{2R}}\right).$$

**Figure 4:**
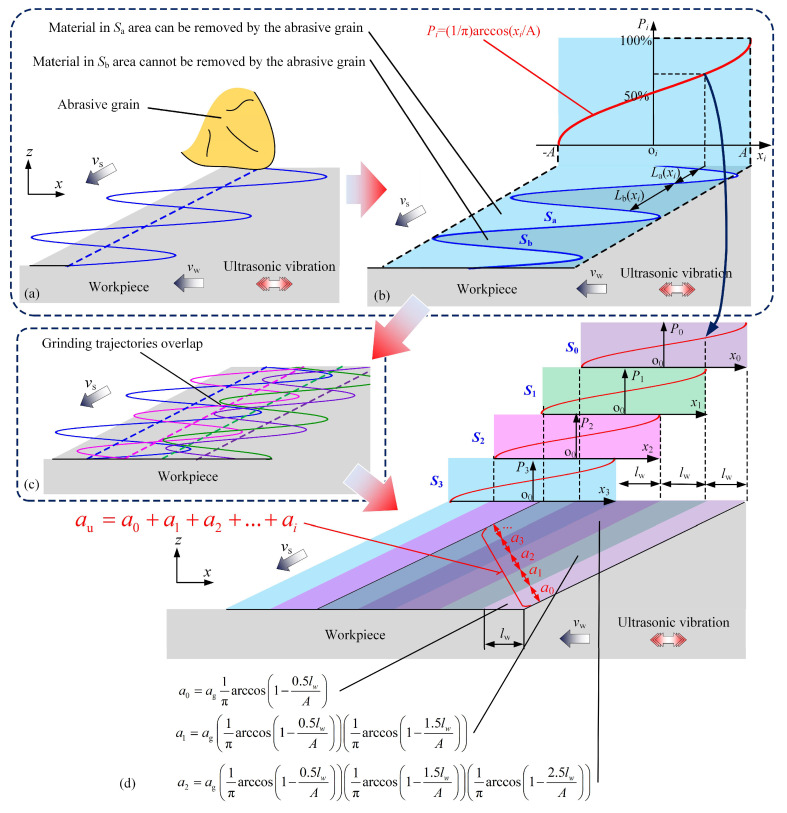
Illustration of undeformed chip thickness in UVG: (**a**) grinding trajectories of abrasive grains in UVG; (**b**) probability curve of material removal; (**c**) grinding trajectories overlap in UVG; and (**d**) probability superposition of material removal area.

Figure 4 illustrates the model of undeformed chip thickness in UVG. The grinding trajectory is changed to a periodic wave curve when the ultrasonic vibration is active, as shown in Figure 4a,b. Furthermore, the grinding trajectories overlap with each other when multiple abrasive grains are considered, as illustrated in Figure 4c. This phenomenon increases the complexity of the undeformed chip thickness.

In the study, the distribution of abrasive grains on the wheel surface follows the uniform probability distribution [33,34] with the unchanged probability density of $1/l_{g}$. The probability superposition method is utilized for the undeformed chip thickness. It considers the material removal process in UVG as vibration area superposition. For instance, for a single abrasive grain, the periodic wave curve covers a vibration area in the grinding zone, as shown in Figure 4b. The material in the vibration area may or may not be removed. In detail, the material in the $S_{a}$ sub-area can be removed by the current abrasive grain; however, the material in the $S_{b}$ sub-area cannot be removed due to the periodic wave curve of the grinding trajectory in UVG, as shown in Figure 4b. A function can be used to describe the relationship between the material coordinate $x_{i}$ and the material removal probability, as follows:

(11) $$P_{i}(x_{i})=\frac{L_{b}(x_{i})}{L_{a}(x_{i})}=\frac{1}{\pi }\arccos\left(\frac{x_{i}}{A}\right),$$

where $P_{i}$ denotes the probability that the material cannot be removed by the abrasive grain. It indicates that the material at the inner position has a low probability of being removed. When the position of the material exceeds the rightmost position of the vibration area in Figure 4b, the probability $P_{i}$ equals 100%. On the contrary, the outer positioned material has a lower probability $P_{i}$. The material must be cut off ($P_{i}=0$) when it is located on the left of the vibration area.

As the trajectory overlap occurs, the grinding zone can be divided into many sub-areas. For instance, the distribution of sub-areas is determined by the superposition of four vibration areas, *S*_0_–*S*_3_ in Figure 4d. The distance between the edges of the sub-areas equals the value of $l_{w}$. The average undeformed chip thickness in UVG au equals the sum of the equivalent undeformed chip thickness in every sub-area, which can be described as follows:

(12) $$a_{u}=a_{0}+a_{1}+a_{2}+\ldots +a_{i},\;(i=0,\;1,\;2\text{…},-1\leq \frac{A-il_{w}}{A}\leq 1)$$

(13) $$a_{i}=\left(P_{i}\times P_{i-1}\times P_{i-2}\times \ldots \times P_{0}\right)a_{g},$$

where $a_{i}$ and $P_{i}$ denote the equivalent undeformed chip thickness and material removal probability of abrasive grain *i*. The expression of $P_{i}$ can be determined based on Equation (11), which can be further written as follows:

(14) $$P_{i}(x_{i})=P_{i}(A-(i+0.5)l_{w})=\frac{1}{\pi }\arccos\left(1-\frac{(i+0.5)l_{w}}{A}\right).$$

According to Equations (12)–(14), the undeformed chip thickness in UVG can be obtained as follows:

(15) $$a_{u}=a_{g}\sum_{n=0}^{m}\left(\prod_{i=0}^{n}\left(\frac{1}{\pi }\arccos\left(1-\frac{(i+0.5)l_{w}}{A}\right)\right)\right).\;(0\leq m\leq \frac{2A}{l_{w}})$$

The contact length and contact rate in UVG are obtained according to Equations (2), (3) and (15). All the units in the abovementioned equations follow the International System of Units.

#### 3.1.2. Evaluation of Intermittent Cutting

**Figure 5:**
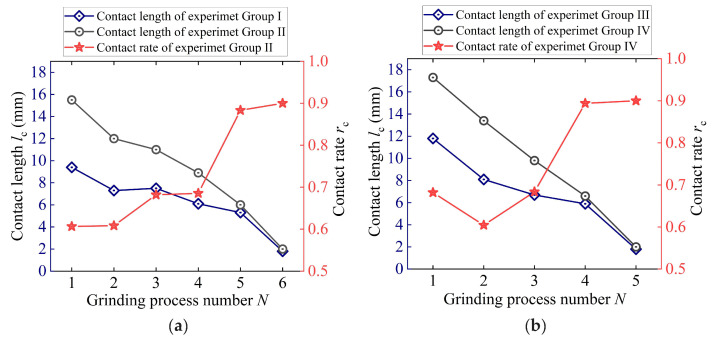
Contact length and contact rate of intermittent cutting behavior under parameters of (**a**) Group I and II and (**b**) Group III and IV.

Figure 5 shows the contact length and contact rate of the experiments of Groups I–IV. Generally, the contact length both in CG and in UVG is reduced with the increase in the grinding process number. The contact rate shows the opposite tendency. In Group I, the contact length in CG reaches the maximum value of 15.5 mm in the first grinding process due to the large grinding depth. The ultrasonic vibration causes a reduction in the contact length to 9.4 mm. The contact rate of UVG is 0.61. The short contact length indicates a remarkable intermittent cutting behavior. With the reduction of the material removal rate, the contact length in CG decreases to 0.2 mm in the last grinding process, and the contact rate increases to 0.9. This suggests that the degree of intermittent cutting behavior becomes limited when the grinding depth is small. The ultrasonic vibration performs better in grinding with large material removal rates.

In Group III, the slow grinding speed of 15 m/s in the first grinding process results in a large contact rate of 0.68. Afterwards, the contact rate firstly decreases to 0.60 and then increases to 0.90. The minimum contact rate is obtained in the second grinding process. The degree of the intermittent cutting behavior becomes weak as the contact rate reaches 9.0 in the last grinding process. This result is consistent with that of Group I. In process 2, the decrease in contact rate is mainly due to the increase in the grinding speed. According to Equation (10), the grinding speed is negatively correlated with the undeformed chip thickness in CG. Namely, the increase in grinding speed leads to a decrease in $a_{g}$, resulting in a reduction of the contact time and contact length. Even though the grinding depth in process 2 decreases to 0.45 mm, which can increase the contact rate to some extent, the grinding speed has more influence on the contact rate than the grinding depth. As a result, the ultrasonic vibration causes the intermittent cutting behaviors under the condition of all the grinding parameters in this study.

### 3.2. Grinding Force

**Figure 6:**
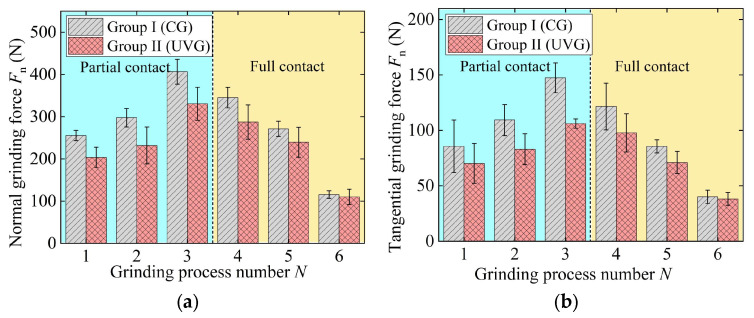
Grinding force in experiment Group I and Group II: (**a**) normal grinding force and (**b**) tangential grinding force.

In Groups I and II, the grinding forces in CG and UVG initially increase and then decrease, reaching a maximum value in process 3, as shown in Figure 6. The maximum normal and tangential forces in CG are 406.40 N and 147.37 N, respectively. The maximum forces in UVG are 18.7% and 27.9% lower than those in CG, respectively. In the first three processes, although the material removal rate gradually decreases, the grinding wheel contact width increases according to the tenon profile, causing the grinding force to rise. The cumulative grinding depth of the first three processes was 1.26 mm, which exceeds the blade tenon profile depth of 1.11 mm. Therefore, in process 4, the grinding wheel is at the full contact stage. The grinding force decreases as the material removal rate declines gradually.

**Figure 7:**
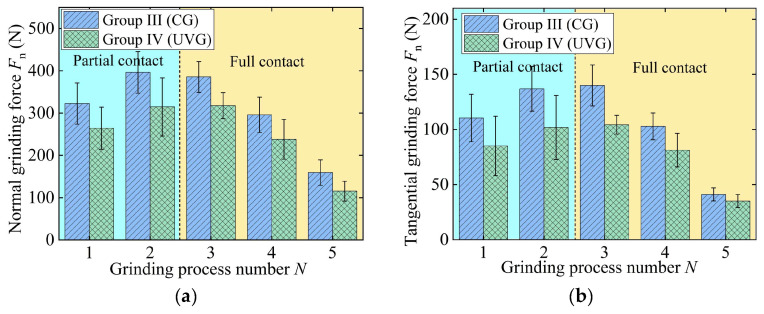
Grinding force in experiment Group III and Group IV: (**a**) normal grinding force and (**b**) tangential grinding force.

The grinding parameters are characterized by large material removal rates in Groups III and IV, wherein processes 1 and 2 are at the partial contact stage and processes 3–5 are at the full contact stage, as shown in Figure 7. As a result, the grinding force in the first two processes increases, while the force in the last three processes gradually decreases, as shown in Figure 7. The maximum normal and tangential forces in CG are 396.32 N and 136.87 N, respectively. The maximum forces in UVG are 20.70% (normal force) and 24.73% (tangential force) lower than those in CG.

**Figure 8:**
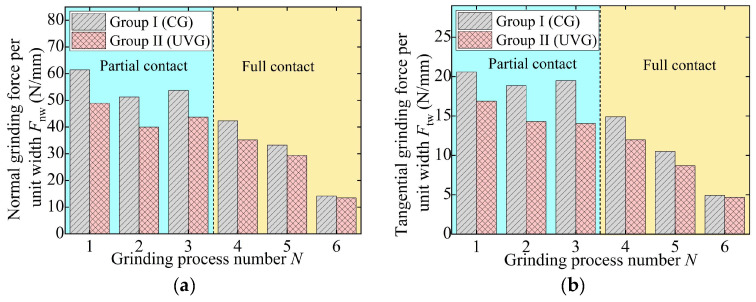
Grinding force per unit width in experiment Group I and Group II: (**a**) normal grinding force and (**b**) tangential grinding force.

**Figure 9:**
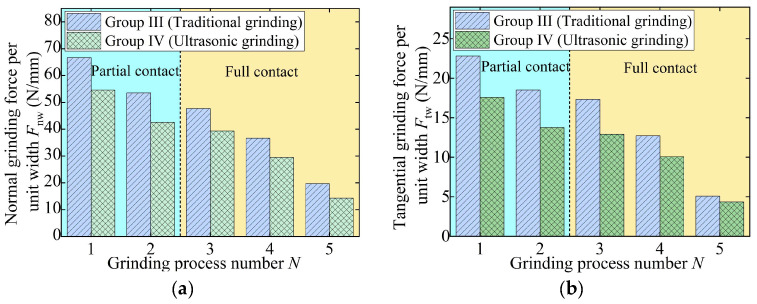
Grinding force per unit width in experiment Group III and Group IV: (**a**) normal grinding force and (**b**) tangential grinding force.

To eliminate the influence of the grinding width on the grinding force, the performance of UVG is evaluated by the grinding force per unit width, as shown in Figure 8 and Figure 9. At the partial contact stage, the normal and tangential forces per unit width in CG remain approximately 53 N/mm and 20 N/mm, respectively. After entering the full contact stage, the grinding force per unit width gradually decreases to 33.25 N/mm and 10.49 N/mm. In the fine grinding stage, due to the low material removal rate, the normal and tangential forces in CG are reduced to 14.16 N/mm and 4.91 N/mm, respectively.

For Groups III and IV, the normal grinding forces in the partial contact stage decrease from 66.65 N/mm (CG) and 54.61 N/mm (UVG) to 53.56 N/mm (CG) and 42.51 N/mm (UVG), respectively. The tangential grinding forces follow a similar trend, indicating that the grinding force gradually decreased throughout the entire process, which facilitates the removal of the grinding-affected layer [35]. As a result, the normal and tangential grinding forces in Group I can be reduced by 15.68% and 18.56% on average, respectively, due to the use of ultrasonic vibration. In the case of Group IV, the normal and tangential forces in UVG are 20.66% and 21.90% lower on average than that in CG, respectively.

### 3.3. Grinding Temperature

**Figure 10:**
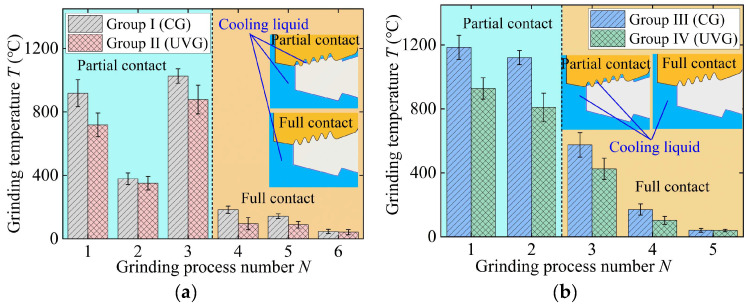
Grinding temperature in (**a**) Groups I and II and (**b**) Groups III and IV.

In Groups I and II, the grinding temperatures in process 1 and process 3 are higher than those in the other four processes, as shown in Figure 10a. This is because the material removal rate in process 1 is the largest. The material generates significant heat during the plastic deformation process, raising the grinding temperature to 918 °C. In the third grinding process, the full contact stage starts, and the continuous and complete contact surface hinders the grinding fluid from entering the grinding area, significantly reducing the heat exchange efficiency. Furthermore, the entire tenon tooth surface becomes a heat-generating area during material removal, limiting the space available for heat conduction when the grinding wheel is fully in contact, resulting in the highest grinding temperature of 1026 °C.

In UVG, the maximum temperature is 877 °C, which is 15% lower than that in CG. The intermittent cutting method of ultrasonic vibration reduces the abrasive grain sliding distance and consequently lowers heat generation. Additionally, the periodic separation between the abrasive grain and material disrupts the coolant laminar flow, enhancing heat exchange efficiency.

In the case of Groups III and IV, the variation of grinding temperature follows a similar trend to the grinding force per unit width; namely, the temperature gradually decreases with the increase in process number, as displayed in Figure 10b. Since the material removal rate in process 1 in Groups III and IV is larger than that in Groups I and II, the grinding temperature in Groups III and IV is relatively higher (1184 °C in CG and 921 °C in UVG). On average, the use of ultrasonic vibration can reduce the grinding temperature by 23.22% in Group II and 23.57% in Group IV.

### 3.4. Workpiece Surface Integrity

#### 3.4.1. Workpiece Surface Topography

**Figure 11:**
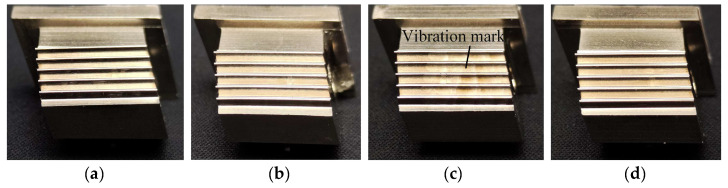
Blade tenons in experiments (**a**) Group I, (**b**) Groups II, (**c**) Group III and (**d**) Group IV.

A high grinding temperature further affects the blade tenon surface, as shown in Figure 11. The blade tenon surface of experiment Groups I, II and IV is smooth. No obvious machining defects can be directly observed. However, visible vibration marks are found on the surface of experiment Group III. This is because the temperature-affected layer of the material is too deep in Group III, and subsequent processes cannot fully remove it, which significantly reduces surface quality.

**Figure 12:**
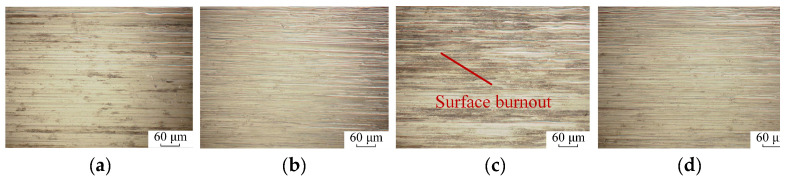
Tenon surface topography in experiments (**a**) Group I, (**b**) Group II, (**c**) Group III and (**d**) Group IV.

Figure 12 shows the surface topography of the K4002 nickel-based superalloy blade tenon. Obvious grinding marks are visible in Groups I, II and IV. However, no intermittent grinding marks are observed on the surface in Groups II and IV, primarily because the grinding depth in the fine grinding process is very small. The small grinding depth of the last grinding process covers the intermittent cutting marks. Moreover, grinding burnout defects are observed on the surface of Group III, as displayed in Figure 12c.

**Figure 13:**
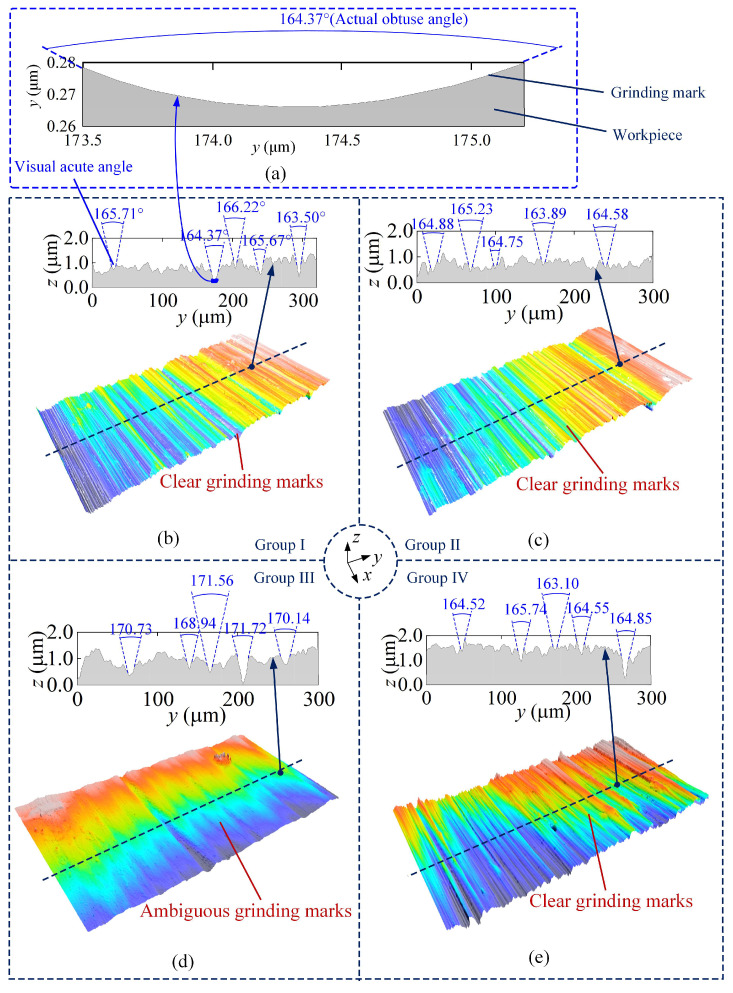
*y*-directional surface topography of ground blade tenon (**a**) illustration of grinding mark under same scale of *z*-axis and *y*-axis, (**b**) surface topography and vertex angles of the grinding marks in Group I, (**c**) surface topography and vertex angles of the grinding marks in Group II, (**d**) surface topography and vertex angles of the grinding marks in Group III and (**e**) surface topography and vertex angles of the grinding marks in Group IV.

The profile curve was then extracted along the *y*-axis, as shown in Figure 13. Clear grinding marks can be found on the surfaces of Groups I, II and IV, while the surface of Group III exhibits ambiguous grinding marks. Only very deep griding marks can be recognized on the surface. The primary reason for this is the high grinding temperature, which causes material thermal softening and subsequent recovery. Under the high temperature, the material can be easily squeezed by the two sides of the abrasive grain instead of generating chips in the material removal process. Therefore, the shallow grinding marks are covered by the squeezing of the material. In other groups, chips can be generated due to the low temperature and poor thermal softening effect, leaving clear marks on the workpiece surface.

To evaluate the workpiece surface quality, 30 grinding marks with clear edges were extracted from the ground surface of each group, and the vertex angles of the grinding marks were measured. The average vertex angles of the grinding marks in Groups I, II and IV are 164.97°, 164.09° and 165.45°, respectively. The standard deviations of measurement are less than 2.5°. However, the average vertex angle in Group III expanded to 170.49°, which is larger than the values of the other groups. Note that all the measured vertex angles are greater than 90° in Figure 13. But the vertex angles are shown as acute angles in Figure 13b–e because the scale of the *y*-axis is much larger than the scale of the *z*-axis. The vertex angle is changed to the correct visual obtuse angle when the scales of the *z*-axis and *y*-axis are under the same order of magnitude, as shown in Figure 13a.

#### 3.4.2. Workpiece Surface Roughness

**Figure 14:**
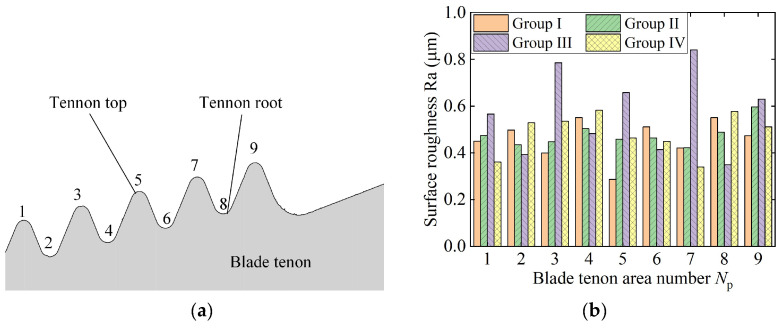
Workpiece surface roughness in different positions of blade tenon: (**a**) area number on the blade tenon surface and (**b**) workpiece surface roughness value.

In the experiments, the surface roughness was measured at nine different positions based on the tenon profile shape. The measured position number is illustrated in Figure 14a, wherein the odd-numbered positions refer to the top of the tenon profile, and the even-numbered positions refer to the root. The results show that the surface roughness in Groups I, II and IV is maintained within Ra 0.4–0.6 µm. The roughness values in the three groups are of same order because the values of the grinding depth in the final machining process are very small ($a_{p}=0.01\;\mathrm{mm}$).

Additionally, the surface roughness in Area 7 of Group III exceeds Ra 0.8 μm. This phenomenon is influenced by the high grinding temperature. The surface roughness of Area 1 is Ra0.57 µm, slightly lower than that of Areas 3, 5 and 7. The shape of the tenon profile affects the transfer of the grinding heat. Area 1 is located at the edge of the tenon, which allows the grinding heat to be easily carried away by the cooling fluid. In the case of the middle areas, the grinding heat is mainly transferred into the material with the heat sources on both sides, resulting in higher temperatures.

The average roughness at the root of the blade tenon (Ra 0.41 µm) is significantly smaller than that at the top (Ra 0.71 µm). This phenomenon is also observed in the other three experiment groups. The root shape of the blade tenon facilitates greater heat transfer into the material than the top shape. Hence, grinding burnout firstly appears at the top of the tenon, making the roughness at the top greater. As a result, the surface integrity of the blade tenon is deeply affected by the tenon profile; the middle area of the tenon has a higher grinding temperature than the edge area because the grinding heat in the edge area can easily transfer to the cooling liquid. Moreover, the top area of the blade tenon is more easily burned out than the root area, due to the inward curved contour. Hence, the middle top area of the blade tenon is most likely burned out on the whole surface of the blade tenon. The use of ultrasonic vibration assistance can improve the surface burnout under the condition of a large material removal rate.

## 4. Conclusions

In this study, four groups of experiments on blade tenon grinding using different process parameters and different methods are investigated. The intermittent cutting behavior, grinding force, grinding temperature and surface integrity are discussed. The main conclusions are listed as follows:

1. The ultrasonic vibration causes intermittent cutting behaviors in the grinding, resulting in the decrease in contact rate to 0.6 at most. The contact rate decreases with the increase in the grinding process, indicating that the effect of intermittent cutting is more significant under the condition of a large material removal rate.
2. For the profile grinding of the blade tenon, the maximum grinding force occurs at the start of the full contact stage. The percentage of the force reduction is negatively correlated with the contact rate in UVG. The grinding force can be reduced by more than 20% on average by the ultrasonic vibration.
3. The grinding temperature varies greatly from larger than 900 °C to below 100 °C throughout the whole machining process of the blade tenon. The ultrasonic vibration assistance can reduce the grinding temperature by 23% on average.
4. For the profile grinding of the blade tenon, the middle top area of the blade tenon has the highest grinding temperature and is most easily burned out. UVG can improve the surface burnout of the blade tenon. The differences of surface topographies and roughness between CG and UVG are inapparent in the fine grinding process because of the large contact rate in UVG under the condition of a small material removal rate.

## Figures and Tables

**Table 1:** Grinding experimental parameters.

Grinding Condition	Parameter
Machine	3-axis profile grinder (Smart BD10, ELB, Germany)
Mode	Down grinding
Abrasive wheel	Corundum and white corundum mixed abrasive grinding wheel (WA/PA80/100F8V35m/sCF3)
Workpiece	K4002 nickel-based superalloy fir-tree blade tenon
Ultrasonic vibration	Amplitude A=5 μm Frequency f=19.3 kHz
Wheel dressing	Diamond roller dresser $\mathrm{Dressing}\;\mathrm{speed}\;v_{\mathrm{ds}}=20\;m/s$ $\mathrm{Dressing}\;\mathrm{infeed}\;\mathrm{depth}\;a_{\mathrm{dw}}=0.3\;\mu m/r$ $\mathrm{Dressing}\;\mathrm{allowance}\;a_{d}=0.2\;\mathrm{mm}$
Cooling medium	5% water-based emulsion (Syntilo 9954, Castro, UK)

**Table 2:** Grinding parameters of experiment Group I and Group II.

Grinding Process Number	Grinding Speed *v*_s_ (m/s)	Workpiece Infeed Speed *v*_w_ (mm/min)	Grinding Depth *a*_p_ (mm)	Ultrasonic Amplitude *A* (μm)	
				Group I	Group II
1	20	150	0.60	0	5
2	20	150	0.36	0	5
Abrasive wheel dressing					
3	20	200	0.30	0	5
4	20	200	0.20	0	5
5	20	300	0.09	0	5
Abrasive wheel dressing					
6	20	300	0.01	0	5

**Table 3:** Grinding parameters of experiment Group III and Group IV.

Grinding Process Number	Grinding Speed *v*_s_ (m/s)	Workpiece Infeed Speed *v*_w_ (mm/min)	Grinding Depth *a*_p_ (mm)	Ultrasonic Amplitude *A* (μm)	
				Group III	Group IV
1	15	150	0.75	0	5
2	20	150	0.45	0	5
3	20	200	0.24	0	5
4	20	300	0.11	0	5
Abrasive wheel dressing					
5	20	300	0.01	0	5

## Data Availability

The original contributions presented in this study are included in the article. Further inquiries can be directed to the corresponding authors.
